# Use of social network sites among adolescents with autism spectrum disorder: a qualitative study

**DOI:** 10.3389/fpsyg.2023.1192475

**Published:** 2023-07-25

**Authors:** Michal Alon-Tirosh, Naama Meir

**Affiliations:** ^1^Behavioural Studies Department, The Max Stern Yezreel Valley College, Tel Adashim, Israel; ^2^Department of Communication, The Max Stern Yezreel Valley College, Tel Adashim, Israel

**Keywords:** autism spectrum disorder, social networking sites, adolescents, communication, subjective experiences

## Abstract

**Background:**

Social network sites (SNS) have become an integral part of the daily lives of billions of users, including adolescents with autism spectrum disorder (ASD). There is a seeming contrast between ASD, characterized by social communication difficulties, and SNS use, requiring social skills. However, few studies examine these adolescents’ personal and subjective experiences on and their self-reports regarding the benefits and difficulties of using them.

**Methods:**

This study examines the communication strategies of adolescents with ASD in using SNS, through semi-structured in-depth interviews with 10 adolescents diagnosed with ASD.

**Results:**

Findings are on three main aspects: reasons for using SNS, actual SNS use, and social characteristics of ASD as expressed through SNS engagement.

**Conclusion:**

The main finding is that SNS use among adolescents with ASD exposes their deficiencies in communication despite providing emotional support. This study highlights the importance of guiding SNS use by adolescents with ASD.

## 1. Introduction

Autism spectrum disorder (ASD) encompasses a wide spectrum of developmental disabilities that affect how a person acts and interacts with others. The symptoms vary among individuals and range from mild to severe (Autism, 2014). According to the Diagnostic and Statistical Manual of Mental Disorders (American Psychiatric Association, 2013), people with ASD exhibit “deficits in social–emotional reciprocity, ranging, for example, from abnormal social approach and failure of normal back-and-forth conversation; to reduced sharing of interests, emotions, or affect; to failure to initiate or respond to social interactions” (p. 50). They also exhibit “deficits in developing, maintaining, and understanding relationships, ranging, for example, from difficulties adjusting behavior to suit various social contexts; to difficulties in sharing imaginative play or in making friends; to absence of interest in peers” (p. 50). Therefore, people with ASD tend to face difficulties understanding situations and other people’s feelings and adjusting their reactions in accordance with the situation. Many also exhibit egocentric self-perspectives, poor social cognition and perceptions, continuous strict and inflexible behaviors, and difficulties sharing enjoyment and self-interest with their social surroundings (Levy et al., 2009; Autism, 2014; Lai et al., 2014).

One of the most discussed characterizations of people with ASD is their difficulty stepping away from their own perspective and understanding a situation from another person’s point of view (Milton, 2014); this is also known as impaired theory of mind (ToM). ToM refers to the ability to understand others’ mental states (e.g., feelings, thoughts, desires, intentions, beliefs) and is considered essential to understanding and predicting their behaviors (Colle et al., 2007). By contrast, impaired ToM is the failure to understand perceptions that are different from one’s own or to see things from another’s point of view. This incapability may transform reality into a series of incomprehensible events and lead to problems understanding others’ behaviors (Baron-Cohen, 2005; Colle et al., 2007). A ToM deficit may be one of the leading factors behind the poor social skills of people with ASD (Happé, 2015).

People with ASD are likely to encounter significant social and emotional difficulties (Mazurek, 2013). Social disabilities are not, however, necessarily characterized by a reluctance to communicate with others (Benford, 2008). In fact, a considerable number of those diagnosed with ASD express a wish to have social connections; unfortunately, their lack of social skills often restricts their ability to express themselves in ways that are understandable and acceptable to others (van der Aa et al., 2016). Nonetheless, despite their social deficits, many people with ASD are attracted to computer-mediated communication (van der Aa et al., 2016) and demonstrate great interest and ability in screen-based media (Mazurek et al., 2012; Mazurek, 2013). They also enjoy similar durations of screen time as those enjoyed by the non-ASD population (Montes, 2016). Past studies have revealed positive relationships between screen-based media usage among people with ASD and their number of friends or social connections, especially with regard to their use of social network sites (SNS) (Benford, 2008; Mazurek, 2013; van der Aa et al., 2016; Sundberg, 2018; Ward et al., 2018).

SNS have become an integral part of billions of users’ daily lives, including people with ASD (Mazurek, 2013). These sites facilitate social interactions among users and enable functions such as sharing and consuming content (Fu et al., 2016). SNS are considered safe environments where users feel in control of their social surroundings (Amichai-Hamburger and Furnham, 2007), and can therefore expand opportunities for people with ASD to communicate with others and improve their offline social networks (Benford, 2008).

Studies have shown that adults with ASD succeed in creating new friendships on SNS and are satisfied with their online social lives (van der Aa et al., 2016). Moreover, adults with ASD who use SNS reported higher levels of happiness than those of adults with ASD who do not, because social interactions on SNS strengthen their social connections (Ward et al., 2018). The frequency of SNS use and the number of online connections of people with ASD were found to be equal, and sometimes even higher, than those of non-ASD individuals; indeed, many people with ASD expressed greater satisfaction with their online social life than with their offline equivalent (van der Aa et al., 2016). SNS have been found to offer people with ASD a unique form of social support, complementing that which is available in their offline lives by enhancing the opportunity to meet others who share similar experiences. This enables communication with peer groups that sometimes do not exist in their offline world, and thus reduces the feelings of social isolation that tend to accompany ASD (Benford, 2008).

Various studies have highlighted the SNS characteristics that are beneficial for people with ASD (Benford, 2008; Mazurek, 2013; Alper, 2014; Gillespie-Lynch et al., 2014; Shane-Simpson et al., 2016; van der Aa et al., 2016; Ward et al., 2018). First, the textual aspects of SNS reduce stimuli, allowing people with ASD to express themselves better than in real-life conversations. Second, SNS communication allows individuals to read messages several times, thereby processing them, understanding them, and composing suitable answers at their own pace; this frees them from the need to produce immediate responses (van der Aa et al., 2016). These advantages could assist people with ASD with their offline communication difficulties by providing them with anonymous and safe environments for practicing their social skills (Benford, 2008).

However, SNS use can also pose challenges for people with ASD (Gillespie-Lynch et al., 2014). The lack of visual cues can weaken their understanding of others’ emotional states or the structural aspects of conversations (Benford, 2008). Additionally, because people with ASD tend to focus on details rather than the larger picture, they may struggle to learn how to use SNS by themselves and the norms accompanying its use and may therefore need guidance on this matter (Burke et al., 2010).

While past studies have examined SNS use by adults with ASD, there has been limited research focusing on the role of SNS in the lives of adolescents with ASD. This may be the result of the dual attitudes of adolescents with ASD and their parents/caregivers toward SNS. SNS provide a platform for adolescents with ASD to communicate and socialize with family and friends. However, parents/caregivers are generally cautious about SNS due to the risk of cyberbullying and inappropriate content (Zhu et al., 2021) as well as the fear that the adolescents will prefer virtual life to “real life” and thus withdraw from the latter (Nath et al., 2016). Consequently, the few studies on this topic have dealt mainly with the difficulties experienced by adolescents with ASD when using SNS and possible interventions to overcome these difficulties and utilize SNSs to improve their social skills and not with other important aspects, such as the needs fulfilled by SNS use (Gwynette et al., 2017; Van Schalkwyk et al., 2017). For example, Van Schalkwyk et al. (2017) discussed the possibility that social media could improve the quality of friendships formed by adolescents with ASD by examining the relationship between social media use, friendship quality, and anxiety levels. They found social media use to be significantly associated with high friendship quality in adolescents with ASD, which was moderated by their anxiety levels. These findings suggest that social media use may contribute to more friendships among adolescence with ASD, which could be attributed to better social skills due to their media use, however, this explanation was not tested. Gwynette et al. (2017) examined whether the use of Facebook improved the social skills of adolescents with ASD. Their rationalization was that since these adolescents use Facebook in any case, it would be beneficial to channel this use for the purpose of learning social skills. However, only six boys and their parents participated in the study and its findings were not statistically significant. It is therefore not possible to reach a conclusion about the effectiveness of the intervention, even if the participants and their parents expressed satisfaction with the intervention. Zhu et al. (2021) found that adolescents with ASD can collaborate in designing a local community group social networking software through an iterative software design process. Their findings suggest that the iteration may promote adolescents’ self-advocacy skills. While the latter two studies look at how social media can be used to improve the skills of adolescents with ASD, they do so by proactively intervening or by using a local social network. They do not examine the adolescents’ experience of use as it actually is, namely, without proactive intervention or the use of existing social media.

Furthermore, most studies concerning adolescents with ASD have relied primarily on the reports of parents/caregivers and have not focused on the personal testimonies of the adolescent users themselves (e.g., Stiller et al., 2019). In other words, the subjective experiences of adolescents with ASD as SNS users have not been sufficiently explored.

Although many adolescents with ASD use SNS, there is a lack of studies examining their personal and subjective experiences and self-reports regarding the benefits and challenges of SNS use (Prendella and Alper, 2022). This study aims to address this gap by examining SNS use among adolescents with ASD and attempting to understand whether and how the communication difficulties characteristic of autism are expressed in their use of SNS. It also investigates whether and how adolescents with ASD engage in social interactions on SNS despite their social limitations.

The study is based on a qualitative methodology that promotes an inductive approach to data collection. This is because the research relates to an innovative subject whose elements and characteristics are not yet known. Qualitative research facilitates a phenomenological examination of participants’ experiences, providing a comprehensive understanding of their subjective perspectives and behavioral patterns. Qualitative research also emphasizes the centrality of participants’ voices and experiences, therefore ensuring that the research captures the authentic realities and needs of the population and thus leading to more meaningful and relevant findings that accurately reflect their perceptions, thoughts, and experiences (Patton, 2002).

The use of semi-structured in-depth interviews enables researchers to explore the intricate nuances of how adolescents with ASD navigate and engage with SNS and capture the essence of their lived experiences. In-depth interviews are based on the relationship created between the interviewer and the interviewee. This relationship enables a deep understanding of the latter’s personal and subjective perceptions while referring to their thoughts, feelings, and experiences. This personal relationship allows the interviewer to be attentive and sensitive to the interviewee’s needs, which can alleviate the communication difficulties faced by adolescents with ASD. The use of semi-structured interviews also offers a flexible data collection method that accommodates the complexity and diversity of the interviewees’ experiences. The use of open-ended questions enables participants to articulate their thoughts and experiences freely while affording researchers the opportunity to delve deeper into specific domains of interest. This adaptability allows for the exploration of emergent themes and ensures that critical topics are not overlooked (Patton, 2002).

## 2. Materials and methods

### 2.1. Participants

Semi-structured in-depth interviews were conducted with 10 adolescents (seven males and three females, aged 15–21 years, average age: 18.6 years) who were diagnosed with ASD and studying in schools belonging to the special education system (designed for children and youth under the age of 21). Adolescents were eligible to participate if they met the following inclusion criteria: (1) they were between 12 and 21 years old (the ages defined as adolescence among children with special needs according to the Ministry of Education in Israel), (2) they were diagnosed with ASD, as reported by their parents, and (3) they had adequate verbal and communication skills to participate in the interviews.

**Figure 1:**
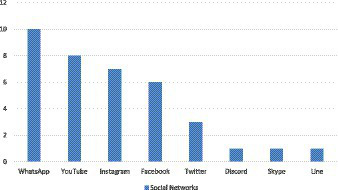
SNS used by the participants.

All participants reported using at least one SNS (M = 3.6 years, SD = 1.22, range: 1–5). Figure 1 shows the SNS used by the participants.

### 2.2. Procedure

Participants were recruited with the help of youth club counselors for adolescents with special needs. Freely given, informed consent to participate in the study was obtained from the youth clubs’ management, adolescents, and their parents, after which dates for the interviews were coordinated. Interviews were conducted at times and places chosen by the participants to provide them with a comfortable setting and enable open discourse (mostly at the participants’ homes, often in their bedrooms). Before the interview, parents signed a consent form for their children’s participation, and the adolescents verbally consented to participate.

At the beginning of each interview, the research objectives were presented to the participants, who were also assured of full confidentiality of their personal details. Any personal stories they were willing to share with the researcher but did not want to reveal publicly were not included in the research. Each interview lasted between one and 2 hours, depending on the participant’s cooperation and patience. A total of 11:45 h of data were collected. An interview guide designed for the current study was used to ensure that the same basic lines of inquiry were pursued with each participant (Patton, 2002); however, between planned questions, the interview was conducted as an open conversation. The main topics covered the participants’ use, experiences, and perception of SNS in their everyday lives.

Questions included: “Do you use SNS? If so, which SNS do you use?”; “Why did you choose to use these SNS?”; “What other SNS do you know? Why are you not using the SNS you specified?”; “What use do you make of SNS? (e.g., Is it creative use? Writing content? Sharing? Likes? Comments? Reading without commenting? Posts? Photos? Live? Messages?)”; “When and where do you use SNS? (Both in terms of physical locations/times and emotional states)”; “When did you start using SNS and under what circumstances did you start using them?”

The complete study protocol was approved by the College Institutional Review Board (removed for anonymity).

### 2.3. Analysis

The interviews were transcribed and then analyzed thematically, in accordance with the conventional procedures of qualitative analysis. A procedure involving repeated readings and refining of categories was applied, aiming at identifying key themes describing the SNS use of adolescents with ASD (Braun and Clarke, 2006; Lindlof and Taylor, 2019). Patterns, concepts, and themes were identified through an intensive process of reading and re-reading the interviews. Each interview was first analyzed separately and then compared with other interviews. The ongoing process of identifying categories employed three types of coding: (1) Open coding: comparing and contrasting initial patterns, thereby uncovering and specifying initial categories, (2) Axial coding: linking initial, related categories to subcategories, and (3) Selective coding: integrating and refining the categories (Strauss and Corbin, 1994). This process resulted in integrating and refining common categories, presented as the main aspects (reasons for using SNS, actual SNS use, and social characteristics of ASD as expressed through SNS engagement) and sub-aspects in the results. Finally, representative quotations were chosen to illustrate the essence of each category and subcategory. As the interviews were conducted in Hebrew, the chosen quotes were translated into English by the authors. Pseudonyms were used to ensure the participants’ anonymity.

## 3. Results

Analysis of the interviews revealed insights on three main aspects: reasons for using SNS, actual SNS use, and social characteristics of ASD as expressed through SNS engagement.

### 3.1. Reasons for using SNS

Three main reasons for using SNS were identified from the interviews: to overcome communication difficulties, to achieve self-disclosure and attention, and to update and coordinate.

#### 3.1.1. SNS use for overcoming communication difficulties

During their interviews, the participants expressed a view that SNS help them cope with, and even alleviate, their communication difficulties. They reported that SNS social interactions assist them in dealing with the language and social difficulties that characterize ASD by providing them with opportunities to “practice” their deficient social skills and improve their language skills. They viewed such interactions as social opportunities and described the contributions of these interactions to their social lives. Participants also noted that SNS interaction enables them to overcome speech impediments that they experience in offline communication.

For example, Avi, who struggles with continuous speech, said:

Writing is easier for me….When I speak face-to-face, I need time to answer the questions, and this makes people move on….Corresponding in writing is easier because I feel like people manage to understand what I’m trying to say.

Similarly, Nir stated:

I started to use SNS two years ago when I was frustrated and didn’t have the courage to communicate with others in the real world…. I had a lack of communication with real people, and this is what SNS exists for.

Nir’s words suggest that his SNS use complements the social deprivation he experiences in the offline world. He described his difficulty connecting with people in his offline life and SNS serving as a desirable solution.

#### 3.1.2. SNS use for self-disclosure and attention

The participants described their desire to expose themselves to others on SNS and thus present their lives. They indicated various types and levels of self-disclosure such as posting photos and even sharing personal feelings with others. They expressed their wish to get others’ attention and be present and meaningful in others’ lives by sharing their feelings. Such attention excites them and makes them feel good.

For example, Shani said: “I post statuses on WhatsApp… It’s important to me to know who saw it; I need to know who saw that I have a life… My last status had 24 views [laughs excitedly].” Her words highlight a fundamental need for self-disclosure. She wants other people to see what happens in her life—that “I have a life”—that is, she has an active personal life. Her excited laugh on noting the number of people who viewed her status emphasized her positive feeling about self-disclosure.

#### 3.1.3. SNS use for updating and coordinating

The participants see SNS, especially WhatsApp, as a platform for communicating with their parents, teachers, members of the educational staff, and friends to coordinate meetings and events, update their schedules, receive updates about necessary equipment, and update others when they are late or absent from an activity. They noted that their communication in WhatsApp groups is mainly for these purposes, with social discourse occupying a very marginal place. Some participants shared that they do not read content that is not related to updates and matters of coordination. Gal, for example, noted: “WhatsApp is important because sometimes important messages should be seen.” Shani expressed her frustration that when she did not have WhatsApp, her life was disrupted because she was unable to remain updated: “There was a week when I did not have WhatsApp, and it actually messed up my life.”

### 3.2. Actual SNS use

The second aspect of the findings was participants’ actual use of SNS, which was found to be largely technical and lacking intimacy. Whereas some used SNS functions correctly, succeeded in having social interactions, and chatted with others who were unaware or oblivious to their disabilities, most participants often misunderstood social codes, expressed a need for guidance, and relied on complementary communication tools. Their SNS use also demonstrated a lack of ToM.

#### 3.2.1. Technical use

Participants seemed to view their SNS interactions and use in a very technical way and, therefore, not primarily for a social purpose. Some described SNS as a tool for storing their photos to avoid filling the storage capacities of their mobile phones. Others noted that their reason for joining a particular SNS was their desire to play certain games or to be exposed to and learn other languages. Still others saw a benefit in SNS in allowing them to maintain contacts and not “lose them,” but also explained that they initiated social interactions only when required. For example, Eden sees SNS as a technical platform that allows her to gather and keep her list of friends. Although a social motive is evident, she nonetheless sees SNS as a telephone directory: “I like that I can save my list of friends in WhatsApp… so that, in the future, they will not disappear, and they’ll still be in my phone.”

#### 3.2.2. Lack of intimacy

Participants clarified that they do not create intimacy when using SNS. They distinguished between online and offline (face-to-face) conversations with their friends and expressed the lack of proximity they generally feel on SNS; deep conversations, they explained, are reserved for offline interactions, whereas SNS conversations comprise relatively superficial content, exchanges of funny messages, or discussions about “light” topics. They noted that they failed to establish closeness in these relationships and, even with their closest online friends, struggled to discuss issues that they felt comfortable talking about face to face. Gil expressed his view that online conversations are not very deep: “The connections on WhatsApp are not real. It’s not a real conversation. Even if it turns out to be a heart-to-heart conversation, it still will not be a real conversation.” Similarly, Nati seemed to have a clear preference for holding personal and intimate conversations offline: “If it’s about something personal that is important to me, I’d rather feel like they are standing next to me so that I can talk to them.”

#### 3.2.3. Misunderstanding social codes

Participants revealed that adolescents with ASD often misunderstood social codes that are norms in SNS use and, therefore, often unintentionally share content in ways that are not socially acceptable. Yair reported: “I sometimes ‘like’ my own photos… I like the way that my photos came out, so I flatter myself [by pressing ‘like’… What? Everybody ‘likes’ their own photos, do not they?]” His words indicate that he did not understand a very basic social code on SNSs, namely, that “liking” one’s own photos is not deemed socially acceptable; although he had been using Facebook for quite a long time, he still failed to understand this social convention.

#### 3.2.4. Need for guidance

Statements about the social rules and conventions of SNS indicated that participants were often unaware of these norms until these rules and conventions are explained to them. Similarly, some situations they described facing on SNS illustrate that they did not know how to act in those situations. Shani, for example, noted:

One time, I chatted with a guy from my class, and he sent me three emojis, but I didn’t know how to answer him… There are some norms that I’m not aware of, and I don’t know if I’m supposed to respond or just move on. Since there is no question mark, I don’t know if I’m supposed to answer.

Her words illustrate a situation wherein she did not know whether she needed to answer and, if so, how, because of the lack of correct punctuation. For her, a question mark is an unambiguous marker, and in its absence, she did not know how to act.

Gil offered a similarly clear example of his need for guidance:

When I got my first smartphone, I sent others everything that was on my mind…. There was one time that I did this foolish thing and put all my WhatsApp contacts in one group, including my transport escort… My father didn’t like this… he said it was the wrong thing to do.

Without a conversation with his father, Gil was unable to understand that he had done something “wrong”; he needed guidance to learn that his actions were not socially acceptable.

#### 3.2.5. Use of complementary functions

When using SNS, participants often had trouble understanding the intentions of the person with whom they were interacting because of the lack of nonverbal cues. They therefore attempted to compensate for these disadvantages by using complementary communication tools such as video chats, Skype, and Facebook or Instagram live broadcasting alongside their SNS interactions. For example, Gil said: “It’s important to add emojis to WhatsApp messages… It’s better to do it, so you can better know how the other person feels.” He seemed to be aware of his problem understanding SNS interactions and found a partial solution by sending emojis, which complemented the understanding and explanations offered in the words of his messages.

#### 3.2.6. Lack of ToM

The participants’ SNS use reflects their lack of ToM: namely, they have difficulty seeing things from others’ perspectives. They could not quite grasp that others might not have understood their messages at some point in the interaction and often failed to comprehend others’ intentions if not explicitly stated. When asked how they were able to infer if other people had understood them properly on SNS, responses included: “If they read my message… If they answer me back” (Eden); “Because they see very well everything I write” (Avi); “I just know” (Yair). Such reactions indicate that these participants saw no possibility that others could have misunderstood them simply because the people they were communicating with had read the messages and responded; they thus perceived reading and answering as understanding. When Gil was asked how he thought another person might have felt when they sent a message to a WhatsApp group and no one replied, he said, “It would not have bothered him… I would not be bothered if it happened to me.” He did not seem to expect the feelings of others to be different from his own and assumed that if he would not be insulted, then neither would others.

### 3.3. Social characteristics of ASD as expressed through SNS engagement

The third aspect of the findings was on the social characteristics of ASD as expressed through SNS engagement. These characteristics include the interest of adolescents with ASD in making social connections despite the difficulties they encounter in making them; awareness of their own communication difficulties; a focus on themselves over others; and the desire to discover new social worlds and be free from the specificities of ASD. In addition, the findings indicated that it is easier for adolescents with ASD to communicate with adults or authority figures than with their peers and that they rely on their families, rather than their friends, as sources of emotional support.

#### 3.3.1. Interest in making social connections despite difficulties

Participants described SNS communication as a positive and significant aspect of their lives and showed their appreciation for its presence. Nonetheless, they expressed difficulties in engaging in SNS and the frustration that sometimes accompanied their efforts. Whereas social interest and the desire to have social connections were evident in every interview, participants expressed that they had problems initiating interactions; they explained that they often sent messages only when they had a concrete reason (e.g., wishing a friend a happy birthday or inviting someone over). Some said that they engaged in SNS conversations only when they were free to do so and not as a regular habit. They also noted that they would often not interact with a friend that they had already met earlier in the day.

Some shared that they began to use SNS not by their own initiative, but by their parents’ or teachers’. For example, when asked about the first time he chose to use WhatsApp, Gal explained:

I didn’t choose… My parents got me my first smartphone because my teacher asked them to. It was important to him that I’d have WhatsApp too, so I could see the updates from school… At first, my friends weren’t the reason why I used it… but when I started communicating with my friends, it felt good to talk to them over the phone for the first time.

It was thus Gal’s teacher who connected him to SNS for technical reasons. Gal’s subsequent enjoyment of SNS communication with his friends indicates his social interest.

Similarly, Dan described using SNS to chat regularly with his close friends:

I do it almost every week if my phone is charged. If it isn’t, then we don’t chat…. I have another friend I’d like to chat with… we don’t chat because I don’t have his phone number [thinks for a few moments silently] … In fact, I’ll do it… I’ll just do it. I’ll just get his number and we’ll chat on WhatsApp… WhatsApp is a great idea… It would be nice to talk about each other’s interests.

Dan expressed his desire for social connection; his need for someone to talk to gave him the idea of chatting with his friend. The fact that he only chats when his phone is charged (and he does not keep it charged) suggests that SNS communication is not an integral part of his life.

#### 3.3.2. Awareness of their own communication difficulties

During their interviews, participants made statements that indicated their awareness of their ASD diagnosis and related difficulties. They described their struggle engaging in social interactions on SNS as one of the ASD characteristics that made them feel different. Yair reported: “I do not know if I’m at a really high level socially… I still have social difficulties… I do not understand normal people socially. I’m on the spectrum, and people with ASD have social difficulty.” Similarly, Gil said: “Compared to normal people, we are few… People who do not have ASD… for example, people who are normal lIke you [pointing at the interviewer], no offense… I do not know if we are smarter than them or they are smarter than us.” His words demonstrate awareness of his problem and the distinction he draws between “us,” that is, people with ASD, and “them,” that is, people he calls “normal.” He perceived this differentiation as fundamental while trying to identify which of the two groups is smarter.

#### 3.3.3. Focus on themselves over others

Descriptions of their SNS use suggested that participants are more interested in themselves than in others. This self-interest was expressed both in the content they choose to share and the way they respond to others on SNS. They explained that the photos they shared and the messages they send are always related to their own interests, with no consideration for whether others will find the content interesting. Likewise, they respond to others’ photos or messages mostly when they find them interesting or when they connect these messages to their own interests; they appeared reluctant to waste time reading content that was unrelated to them. For example, when asked about his SNS interests, Dan responded:

I’m interested in content related to video games, building worlds, languages… practical issues, interests. Not people. Maybe I’m a little interested in politics, but not in people. I’m not into gossip; I’m not interested in content posted by my friends, unless it suits my interests.

Similarly, Avi said:

There was a time when I was a member of my family’s WhatsApp group. I used to send my paintings, and they would compliment me and say they were beautiful and amazing and that I’m talented… I left the group because they started to send a lot of messages and photos. It filled up my phone storage.

#### 3.3.4. Desire to discover new social worlds and be free from the specificities of ASD

Participants described the different attitudes of SNS users who knew of or noticed their disability and expressed their preference for others to be unaware of their disability to avoid being treated differently. Whereas face-to-face social interactions involve physical disclosure and the potential for others to notice their difference, SNS allow for social interactions without the demand to reveal real identities and offer the opportunity to present desirable pieces of personal information that the participants are happy to share with others. Therefore, SNS enable adolescents with ASD to engage in social interactions without being defined according to ASD or special-needs categories, to get out of their familiar world, remain anonymous, and have new relationships with new beginnings. As Yair said: “It’s much easier to communicate on WhatsApp because of the screen… others cannot tell how I act in reality.”

Likewise, when Nir was asked if he preferred not to disclose on SNS that he studied in a special education school, he responded:

I’d rather keep it to myself… because there’s always some possibility that they will laugh… that they won’t be interested, or will just say things about me…. I don’t want people to be careful in my presence… I want people to be free to talk about anything they want when they are next to me without being afraid of hurting me.

Here, he described two types of reactions that he receives from others and prefers not to experience: insulting and overcautious. Both attitudes stem from defining him as a “person with ASD”—a situation of which he is well aware. He believes that by not exposing his ASD identity on SNS, he can prevent these unwanted reactions.

#### 3.3.5. Preference for communication with adults or authority figures than with their peers

Participants indicated that it was easier for them to communicate with adults or authority figures than with their peers. They reported sending more SNS messages to those responsible for them than to their friends and preferring to chat in SNS groups where staff members are present. Some noted that when they wanted to meet a friend, they often called their friends’ parents rather than calling their friends directly; some even explained that they might contact a friend to get their parent’s phone number and then talk to the parent to arrange the meeting. For example, Eden said: “I called my friend’s mom… because I wanted her daughter to visit me… Usually, you need to coordinate with the parents before you coordinate with their child. I have done this with her mom for years.”

#### 3.3.6. Relying on family rather than friends as a source of emotional support

Most of the participants explained that only their parents could really help them and thus they turned to their parents, and not to their friends, when they needed support. Nati, for example, explained:

If I’m in a bad mood, I’ll probably open YouTube and watch some videos, and if I still feel bad, I’ll talk to someone… I’d probably talk to my mom… because I know she usually has ways to help me. I mean, I don’t know how my friends could help me with my mood.

Similarly, when Gil was asked if he talks to his friends when he is sad, he said: “When I’m sad?… Usually, on such issues, my parents help me. My friends do not help me.”

## 4. Discussion

This study aims to identify the communication strategies of adolescents with ASD when using SNS. It addresses the lack of studies examining the personal and subjective experiences and self-reports of adolescents with ASD regarding the benefits and challenges of SNS use and looks to understand whether and how the communication difficulties characteristic of ASD are evident in their use of SNS. The study also investigates whether and how adolescents with ASD engage in social interactions on SNS despite their social limitations. Accordingly, semi-structured in-depth interviews were conducted with 10 adolescents with ASD. Analysis of the interviews revealed insights on three main aspects: reasons for using SNS, actual SNS use, and social characteristics of ASD as expressed through SNS engagement. This study’s findings align with those reported in other studies on ASD and SNS, including the following: SNS use could assist people with ASD in overcoming their communication difficulties (van der Aa et al., 2016); adolescents with ASD are aware of their communication difficulties (Jones et al., 2003; Benford, 2008); adolescents with ASD are interested in making social connections despite the difficulties they encounter in making them (Benford, 2008; van der Aa et al., 2016); adolescents with ASD tend to focus on themselves (Baron-Cohen, 2005); adolescents with ASD prefer to communicate with adults or authority figures rather than their peers (Howlin et al., 2004; Ingram et al., 2007); adolescents with ASD tend to rely on their families, rather than their friends, as a source of emotional support (Seltzer et al., 2004; Bayat, 2007; Peek and Stough, 2010); and adolescents with ASD need guidance in their SNS use (Burke et al., 2010).

Our findings on the SNS use of adolescents with ASD also correlate with those of other studies on people with ASD but not in the context of SNS use. For example, the inflexibility and “technical” SNS use revealed herein have been found to be consistent with known characteristics of individuals with ASD in different contexts (Georgiades et al., 2007; Burke et al., 2010; Gillespie-Lynch et al., 2014). The tendency to misunderstand social codes while working in an SNS environment is compatible with other findings about the general misunderstanding of social codes by people with ASD (Derks et al., 2007). Lack of ToM has also been reported as a characteristic of people with ASD in other non-SNS environments (Colle et al., 2007).

On the other hand, the reasons for and feelings about using SNSs that were observed in this study among adolescents with ASD have also been reported in studies about SNS use by the general population and not only by people with ASD. These include the following: SNS use for updating and coordinating (Quan-Haase and Young, 2010; Buhler et al., 2013; Froment et al., 2017); a desire for self-disclosure and attention (Mckenna et al., 2002; Amichai-Hamburger and Furnham, 2007); a feeling of lack of intimacy on SNS (Pornsakulvanich et al., 2008; Karr-Wisniewski et al., 2011); the use of complementary functions with SNS interactions (Derks et al., 2008; Inkpen et al., 2012; Buhler et al., 2013; Greenberg and Neustaedter, 2013); joy at receiving feedback on SNS while not grieving its absence (Reich et al., 2018); and the desire to discover new social worlds and be free from specifications (Mckenna et al., 2002; Amichai-Hamburger and Furnham, 2007). However, these aspects were not reported in previous studies focusing on people with ASD. This is possibly because these studies did not examine the experiences with SNS use from the perspectives of people with ASD or because they highlighted the differences in SNS use between the people with ASD and the general population and thus failed to identify similarities in the patterns of use.

The primary motive for conducting this study was to examine if SNS use could significantly help adolescents with ASD overcome their social communication difficulties. Our findings do not, however, provide definitive answers. On the one hand, SNS use among the participants was found to be mainly non-social and revealed participants’ deficiencies in communication. They used SNS mainly for updates and coordination and not for social interaction, and thus their SNS use was very technical. In addition, they found it difficult to understand the basic social codes that are considered norms on SNS and to create intimacy within their SNS social interactions. Furthermore, they showed interest almost solely in themselves and not in others; they faced difficulties understanding the other person in their SNS conversations and imagining that others can think differently from them. These factors affected their SNS social interactions and made it difficult for them to engage in effective social communication. In these aspects, SNS were not found to solve the social deficiencies experienced by adolescents with ASD, particularly as they could not depend on the non-verbal cues that are available in offline interactions.

On the other hand, all the participants expressed positive attitudes toward SNS use and described its importance in their lives. Despite some SNS characteristics and norms that made it difficult for them to form social interactions, they described some essential characteristics of SNS that enabled them to overcome their social difficulties and make SNS a positive space for adolescents with ASD. These included having a platform that allowed them to read another person’s words in a conversation several times, thus enabling them to properly understand the message, word their answers without time limitations, avoid their speech impediments (such as slow speech), and “practice” social interactions to improve their social skills without revealing their identity. These insights left an overall impression that the negative traits of SNS were marginal compared to the positive ones. Participants shared a feeling that SNS use compensated for certain limitations that were characteristic of people with ASD. They regarded SNS as opening up a new and positive world, enabling them to keep meeting their peers and others like them, and allowing them to interact without their ASD label. It can be said that this positive attitude of adolescents with ASD toward SNS corresponds with the feelings of people without ASD toward SNS (Chan-Olmsted et al., 2013; Tsay-Vogel et al., 2018).

One problem that remains unclear is whether SNS are, in some way, “insensitive” to people with ASD. We observed that some participants managed to overcome some of their difficulties, use SNS functions properly, succeed in making social interactions, and even chat with people who were oblivious to their disabilities. However, others experienced social and communication difficulties when interacting via or using SNS, thus transferring their misunderstanding of social codes and non-social communication to their interactions on SNS. It can thus be concluded that whereas SNS allow adolescents with ASD to solve certain challenges (e.g., speech impairments, pace of interaction, feelings concerning their place in society, or feelings of insecurity), these factors do not necessarily enable them to resolve deeper communication problems.

This study’s findings suggest that SNS have a significant place in the lives of adolescents with ASD who attach great importance to interacting via SNS. This usage aligns with the general behaviors and attitudes of the population at large, and perhaps, as a result, SNS use by adolescents with ASD is not perceived as exceptional or pivotal. Nonetheless, some adolescents with ASD find it difficult to understand social norms or to learn behaviors and actions that are regarded as appropriate in various contexts, including SNS; thus, they often misuse SNS and exhibit non-social behaviors in their SNS use. In fact, participants stated that while they use SNS, they sometimes do not know how to use these platforms properly and effectively and have encountered situations where they did not know how to act and needed external assistance. It therefore seems imperative that they receive guidance on SNS social interactions (e.g., the possibility that the other person in their SNS conversation may think differently from them), SNS social codes (such as norms of SNS use, e.g., when and who is it socially acceptable to “like”), and the rules of SNS conversations (e.g., when they are expected to address a question or a statement made by others in their SNS conversation and when they are not). Such guidance is particularly important because SNS are available 24 h a day and are thus a useful venue for adolescents with ASD to learn, practice, and work on the social and emotional problems associated with ASD.

Despite its contributions, this study has certain limitations. First, with regard to the sample, 10 interviews were conducted with adolescents living in the same geographical area, which may signify that they belong to a specific cultural and social group. This is a limitation because the findings may not reflect adolescents who belong to other cultural and social groups and therefore cannot be generalized to adolescents from other groups. For example, the finding that adolescents with ASD prefer to rely on their parents rather than friends as a source of emotional support might be related to a specific cultural and social affiliation and not to being adolescents with ASD. Future studies should examine SNS use using a broader sample encompassing other geographical areas. Second, the study relied on the participants’ self-reports regarding their SNS activities. Additional information could be obtained from examinations of the online content published by this population (and also by an adult ASD population) and analyses of what this content demonstrates about the relationship between the subjective feelings of adolescents with ASD and their actual experience in using SNS.

## 5. Conclusion

This study provides a window for learning about the role of SNS in the lives of adolescents with ASD, allowing us to understand the benefits of SNS for them and the need for external sources to assist them. It aimed to examine the insufficiently studied issue of whether and how the communication difficulties characterizing adolescents with ASD are expressed in their SNS use and, whether adolescents with ASD manage to have social interactions on SNS despite their social deficiencies.

This study is not the first to examine the SNS use by people with ASD. However, unlike previous studies, this qualitative study conducted direct in-depth interviews with adolescents with ASD. The uniqueness of this study lies in the choice to focus on the personal perceptions of these adolescents. Our conversations were with the adolescents themselves in an attempt to understand their personal feelings and experiences, and not with their parents who might offer different perspectives. This study thus emphasizes the importance of research that involves talking directly with adolescents with ASD about both SNS and other issues.

## Data availability statement

The raw data supporting the conclusions of this article will be made available by the authors, without undue reservation.

## Ethics statement

The studies involving human participants were reviewed and approved by the Max Stern Yezreel Valley College ethics committee. Written informed consent to participate in this study was provided by the participants’ legal guardian/next of kin.

## Author contributions

MA-T and NM contributed to the conception and design of the study, data collection and analysis, and manuscript writing. All authors contributed to the article and approved the submitted version.

## Conflict of interest

The authors declare that the research was conducted in the absence of any commercial or financial relationships that could be construed as a potential conflict of interest.

## Publisher’s note

All claims expressed in this article are solely those of the authors and do not necessarily represent those of their affiliated organizations, or those of the publisher, the editors and the reviewers. Any product that may be evaluated in this article, or claim that may be made by its manufacturer, is not guaranteed or endorsed by the publisher.
